# Intravenous immunoglobulin therapy for small fiber neuropathy: study protocol for a randomized controlled trial

**DOI:** 10.1186/s13063-016-1450-x

**Published:** 2016-07-20

**Authors:** Bianca T. A. de Greef, Margot Geerts, Janneke G. J. Hoeijmakers, Catharina G. Faber, Ingemar S. J. Merkies

**Affiliations:** Department of Neurology, School of Mental Health and Neuroscience, Maastricht University Medical Center, P.O. Box 5800, 6202 AZ Maastricht, The Netherlands; Department of Neurology, St. Elisabeth Hospital, Willemstad, Curaçao

**Keywords:** Small fiber neuropathy, Painful neuropathy, Immunology, Intravenous immunoglobulin, Randomized controlled trial

## Abstract

**Background:**

Small fiber neuropathy is the most common cause of neuropathic pain in peripheral neuropathies, with a minimum prevalence of 53/100,000. Patients experience excruciating pain, and currently available anti-neuropathic and other pain drugs do not relieve the pain substantially. Several open-label studies have suggested an immunological basis in small fiber neuropathy and have reported efficacy of treatment with intravenous immunoglobulin. Therefore, immunological mechanisms conceivably may play a role in small fiber neuropathy. To date, no randomized controlled study with intravenous immunoglobulin in patients with small fiber neuropathy has been performed.

**Methods/design:**

This study is a randomized, double-blind, placebo-controlled, clinical trial in patients with idiopathic small fiber neuropathy. The primary objective is to investigate the efficacy of intravenous immunoglobulin versus placebo on pain alleviation. A 1-point change in the PI-NRS compared to baseline is considered the minimum clinically important difference. In the IVIg-treated group, we assume a response rate of approximately 60 % based on the criteria composed by the IMMPACT group for measurement of pain. Based on this, a sample size of 60 patients is needed. Eligible patients fulfilling the inclusion/exclusion criteria will be randomized to receive either intravenous immunoglobulin or placebo (0.9 % saline). The treatment regimen will start with a loading dose of 2 g/kg body weight over 2–4 consecutive days, followed by a maintenance dose of 1 g/kg body weight over 1–2 consecutive days given three times at a 3-week interval. The primary endpoint is the comparison of the percentage of responder subjects between the two treatment groups from the first randomization during the 12 weeks of treatment. A responder is defined as ≥ 1-point Pain Intensity Numerical Rating Scale improvement on the mean weekly peak pain relative to baseline. The secondary outcomes are pain intensity, pain qualities, other small fiber neuropathy-related complaints, daily and social functioning, as well as quality of life. In addition, safety assessments will be performed for adverse events, vital signs, and laboratory values outside the normal range. Responders during the 12-week treatment period will be followed during a 3-month extension phase.

**Discussion:**

This is the first randomized, double-blind, placebo-controlled clinical trial with intravenous immunoglobulin in patients with idiopathic small fiber neuropathy. Positive findings will result in a new treatment option for small fiber neuropathy and support an immunological role in this condition.

**Trial registration:**

ClinicalTrials.gov, NCT02637700. Registered on 16 December 2015.

**Electronic supplementary material:**

The online version of this article (doi:10.1186/s13063-016-1450-x) contains supplementary material, which is available to authorized users.

## Background

Small fiber neuropathy (SFN) is a disorder of the thinly myelinated Aδ-fibers and the unmyelinated C-fibers, with a minimum prevalence of 53/100,000 [1]. Patients suffer from neuropathic pain, usually according to a length-dependent pattern [2]. In addition, they report autonomic symptoms such as palpitations, gastrointestinal disturbances, and orthostatic dizziness [3, 4]. SFN interferes with daily functioning and may lead to a decrement in quality of life expectations [5]. The diagnosis is based on SFN-related symptoms, without signs of large fiber involvement, in combination with an abnormal intraepidermal nerve fiber density (IENFD) in skin biopsy and/or abnormal temperature threshold levels in quantitative sensory testing [3, 4]. Despite intensive search for underlying causes such as diabetes mellitus, impaired glucose tolerance, Fabry’s disease, hereditary disorders, celiac disease, sarcoidosis, HIV, and other systemic illnesses that may be potentially treatable [3, 4], the proportion of patients with idiopathic SFN (I-SFN) remains substantial, ranging in different series from 24 % up to 93 % [1, 6–8]. Conceivably, immunological mechanisms may play a role in patients with I-SFN, as several immune-mediated diseases such as sarcoidosis, Sjogren’s disease, and systemic lupus erythematosus are associated with SFN [8–11]. Autoantibodies have also been reported in patients with SFN [12–14]. Moreover, inflammatory changes in nerves have been found [15, 16]. Elevated proinflammatory cytokines have been suggested to be involved in the pathophysiology of pain in SFN [17]. In other immune-mediated neuropathies such as chronic inflammatory demyelinating polyneuropathy, treatment with intravenous immunoglobulin (IVIg) has proven to be efficacious [18, 19]. Moreover, some immunomodulation therapies have shown efficacy in some open-label case studies in patients with SFN with chronic pain [20–24]. Similar findings have been reported in erythromelalgia, a condition that is associated with SFN [25, 26]. Pain reduction with IVIg treatment has been summarized recently [23].

IVIg is a blood product with high doses of pooled IgG molecules, which are derived from thousands of donors. IgG antibodies are the primary mediators of protective humoral immunity against pathogens but can also be pathogenic [27]. IVIg may be used either to boost the patients' immunological capabilities or, conversely, to blunt an immune response directed toward the patients’ own tissues [28]. This dual IVIg-mediated effect on the immune system makes IVIg suitable for the treatment of several different diseases. When administered in high concentrations, IVIg has anti-inflammatory properties. How this anti-inflammatory effect is mediated has not been fully elucidated yet. Several mechanisms have been proposed, including toxin inactivation, stimulation of the leukocyte and serum bactericidal action, modulation of cytokine effect, and the modulation of the complement system [28].

In SFN, current neuropathic pain treatment options are generally insufficient to relieve the pain substantially [29, 30]. Therefore, a better treatment is warranted. IVIg appears to be a potential therapeutic option for pain alleviation in SFN. The aim of the current pilot study is to investigate the efficacy and safety of IVIg in patients with I-SFN in a randomized, double-blind, placebo-controlled, clinical trial.

## Methods/design

### Objectives

The primary objective of the study is to evaluate the efficacy of IVIg treatment for pain alleviation compared to placebo in patients with skin-biopsy-proven I-SFN.

Secondary objectives are to assess the effect of IVIg on pain intensity, pain qualities, and other small fiber neuropathy-related complaints and daily and social functioning, as well as quality of life. In addition, safety features of IVIg therapy in SFN will be evaluated.

### Study design

**Fig. 1:**
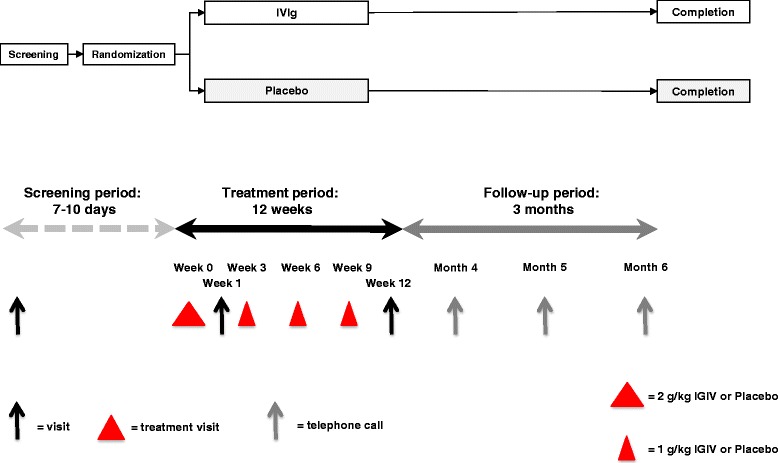
Schematic diagram representing overall study design and study visits Legend: IVIg = intravenous immunoglobulin, red triangles represent the treatment visits. The first treatment visit is spread out over 2-4 consecutive days, treatment visit 2-4 will consist of 1-2 consecutive days

The study has a randomized, double-blind, parallel group, placebo-controlled prospective design, which is shown in Fig. 1. This design has been partly applied and published previously [18, 31]. This design has been partly applied and published previously in the ICE-trial, in which the effect of IVIg for the treatment of chronic inflammatory demyelinating polyradiculopathy has been studied [18]. In that study, a screening period up to 10 days was chosen; thereafter, patients were divided into one of the two parallel groups: IVIg or placebo. Patients received a baseline-loading dose of 2 g/kg over 2–4 days, followed by a maintenance infusion of 1 g/kg over 1–2 days every 3 weeks. For the current trial, this part of the study design has been adopted.

In brief, after a screening period of ≤ 10 days, eligible subjects are randomized to receive either IVIg at an uploading dose of 2 g/kg body weight (bw), ideally over 2–4 (consecutive) days, or a matching placebo. Thereafter, a study drug infusion is administered every 3 weeks at a dose of 1 g/kg bw, given over 1–2 (consecutive) days for a total of three additional infusions following baseline. The subjects’ pain is measured using Pain Intensity Numerical Rating Scale (PI-NRS) at baseline and at each study visit scheduled every 3 weeks for 12 weeks. Pain is also assessed twice a day (daytime and nocturnal pain, through PI-NRS) on 2 days each week (Monday and Friday). A responder is defined as a ≥ 1-point improvement on the mean weakly peak pain using the PI-NRS relative to baseline. Patients who show an improvement and complete the 12 weeks of study treatment will be followed during a 3-month-extension phase to determine the long-term effect of the received therapy on pain alleviation.

The study is placebo-controlled because in previous analgesic trials a placebo effect of 7-37 % has been shown [32]. To make sure IVIg has a factual effect on pain reduction, a placebo-controlled design is necessary to exclude the placebo effect of this treatment on the patients. In addition, patients are allowed to use pain medication that not has been changed in the 30 days prior to randomization. As a result, in case the patient receives a placebo, the treatment does not differ from the situation before participating in the study.

#### Setting and duration

The study is conducted at the department of Neurology of the Maastricht University Medical Center (Maastricht UMC+), Maastricht, the Netherlands. For logistical reasons, all subjects are residents of the Netherlands. The duration of the study is 6 months per subject. Examination will be performed by neuromuscular experts and/or highly trained fellows in neurology. We are aiming to conduct the study in a 2-year period.

### Participants

A total of 60 patients, 30 per treatment arm, will be included in the study. These are patients with newly or previously diagnosed skin-biopsy-proven idiopathic (predominantly) SFN. Subjects are recruited at the Maastricht UMC+, the Netherlands. Informed consent will be obtained from all participants before the study start.

#### Inclusion criteria

Subjects must meet all of the following inclusion criteria to be eligible for enrollment into the study:

1. Participant must be 18 years of age or older.
2. Skin-biopsy proven idiopathic SFN or idiopathic painful neuropathy with a predominant SFN pattern must be present.
3. Pain intensity rated ≥ 5 on the PI-NRS (maximum pain) or on the neuropathic pain scale (NPS) [33, 34], question number 1, must have existed for at least 12 weeks before the study, as declared by each patient to the best of their knowledge; if available, the medical records of each patient will be consulted on the reported pain intensity.
4. Each subject will receive an information leaflet and an informed consent form. Subjects must give written informed consent prior to study entry.
5. Eligible patients must be willing to complete all study-related activities and examination required by the protocol.

#### Exclusion criteria

Subjects presenting with any of the following will not be included in the study:

1. Predominant clinical picture of large nerve fiber involvement (i.e., weakness, loss of vibration sense, and hyporeflexia or areflexia)
2. Treatment with IVIg or any other immunomodulatory/immunosuppressive agents (e.g., steroids) within the last 12 weeks prior to the date of informed consent
3. An underlying cause of SFN (diabetes, *SCN9A/SCN10A/SCN11A* mutations, hypothyroidism, renal failure, vitamin B12 deficiency, monoclonal gammopathy, alcohol abuse (more than 5 IU/day), malignancies, or drugs that cause neuropathy (e.g., chemotherapy, amiodarone, and propafenone))
4. History of anaphylaxis or severe systemic response to immunoglobulin or with a blood product
5. Cardiac insufficiency (NYHA III/IV), cardiomyopathy, significant cardiac dysrhythmia requiring treatment, unstable or advanced ischemic heart disease, history of congestive heart failure, or severe hypertension (diastolic blood pressure > 120 mmHg or systolic > 170 mmHg)
6. Known hyperviscosity, history of renal insufficiency or high serum creatinine levels (MDRD < 30), selective IgA deficiency, or hypercoagulable state.
7. Conditions whose symptoms and effects could alter protein catabolism and/or IgG utilization (e.g., protein-losing enteropathies, or nephrotic syndrome).
8. Females who are pregnant, breast-feeding, or, if of childbearing potential, unwilling to practice adequate contraception throughout the study
9. Mentally challenged adult subjects unable to give independent informed consent
10. Patients using pain medication that has changed in the 30 days prior to randomization (unchanged pain medication is allowed, provided dosages stay equal during the study)

### Study medication

Gamunex© 10 %, 100 mg/ml, solution for infusion is a human normal immunoglobulin that is currently available commercially in a number of countries for the treatment of primary immunodeficiency, idiopathic thrombocytopenic purpura, and chronic inflammatory demyelinating polyneuropathy, as well as other indications in some countries. Placebo is supplied as 0.9 % saline.

The dose of IVIg chosen for this study has been considered to be potentially the most effective in other immune-mediated polyneuropathies, specifically 2.0 g/kg of IVIg as loading dose followed by 1 g/kg bw for maintenance at intervals of 3 weeks [18].

The maximum dose is 80 g IVIg per infusion day, even for subjects whose body weight exceeds 80 kg. The maximum dose is 160 g IVIg for a 2 g/kg bw application and 80 g for 1 g/kg/bw application.

The calculated dose is administered over a 2- to 4-day period at baseline, dividing the total dose equally among the amount of infusion days. The infusion will be prepared on the day of infusion and administered on that same day. The three additional infusions, given at subsequent study visits, are each administered as a single infusion on 1 day but may be given over 2 consecutive days for reasons of tolerability.

On the first 2 days of the treatment (day 1 and day 2), the initial infusion rate will be 0.05 mL/kg/hour for the first 20 min. If no evidence exists of a hypersensitivity reaction, the infusion rate will be increased to 1.0 mL/kg/hour for the next 20 min. After that, the infusion rate will be increased to 3.0 mL/kg/hour. If this is well tolerated, the infusion rate will start at 1 mL/kg/hour for additional treatments and will be increased to 3 mL/kg/hour and 5 mL/kg/hour over 20 minutes up to a maximum allowable rate of 7 mL/kg/hour. This infusion scheme is according to the protocol of the hospital. Each infusion will take approximately 3–4 h. Vital signs will be documented during the infusion. The subject will be monitored during the infusion for any adverse events.

#### Compliance

The volume of the study drug administered will be documented in the medical record and the electronic case report file (eCRF). When less than 100 % of the calculated study drug volume is given, the reasons for deviation will be recorded in the medical record and eCRF.

### Randomization and blinding

All eligible subjects participating in the study will receive a subject number consecutively beginning with the abbreviation of the study (IVIG) followed by 01, 02, or 03, etc. A computer will randomize the subjects to one of the two treatment groups. An automatic message with this allocation will be send to the unblinded pharmacist and will remain confidential.

The study will be subject and investigator blinded during the treatment periods, from visit 2 until the end of the study. Blinding codes will only be broken in emergency situations for reasons of subject safety.

Blinding of different study groups will be guaranteed by ensuring all subjects receiving the same total volume per kilogram of body weight of trial medication, with no visible difference in the external aspect between IVIg and placebo, by using nontransparent infusion lines and bags. An unblinded pharmacist or designee will prepare study medication. This individual, responsible for dispensing the drug, will also be responsible for the blinding procedure.

### Outcome measurements

#### Primary outcome

The primary outcome measure will be based on pain assessment, as pain is considered the most important feature of SFN [2–4, 35, 36]. Pain intensity will be evaluated using the 11-point Pain Intensity Numeric Rating Scale (PI-NRS; 0, no pain, to 10, worst imaginable pain) [33, 37]. In particular, a difference in the mean weakly peak pain intensity will be considered as the primary outcome parameter. A responder is defined as ≥ 1-point improvement on the PI-NRS during the 12-week treatment relative to baseline. The rationale for choosing the primary outcome measure was based on recommendations regarding the clinical importance of treatment outcomes in chronic pain clinical trials as postulated by the IMMPACT (Initiative on Methods, Measurements and Pain Assessment in Clinical Trials) study group [38, 39].

#### Secondary outcomes

Secondary outcome measures include changes in the daily pain intensity, nocturnal pain intensity, and the average of these two obtained from the PI-NRS, change in patients’ global impression of change (PGIC) [38, 39], the Rash-transformed 13-item SFN Symptoms Inventory Questionnaire (RT-SFN-SIQ) [40], the amount of pain medication, the use of nonmedical rescue activities, the amount of pain relief (using a 5-point Likert-scale), the NPS [34], the Daily Sleep Interference Scale (DSIS), the Short Form 36 Health Survey (SF-36) [41, 42], and the Rasch-built Overall Disability Outcome Scale (SFN-RODS) [40].

Safety evaluation features will include the following additional tests: adverse events, laboratory test (e.g., hematology and clinical chemistry, as shown in Table 2), and vital signs.

### Data management

An eCRF is used for each patient to collect all data. To host the eCRF, MACRO electronic data capture is used, powered by InferMed Ltd, London. It has been designed to support compliance with the requirements of relevant regulatory bodies including ICH Good Clinical Practice (www.infermed.com). Assessments start at the screening visit, are subsequently performed according to the scheme presented in Fig. 1, Table 1, and Table 2, and include a standardized interview to determine patient’s clinical condition and well-being, assessment of various questionnaires, and laboratory assessment. During each visit, adverse events and concomitant medication are discussed. At each visit, the diary and residual medications are collected.

Privacy of the patients is guaranteed; stored data and materials are only identifiable to the person by a sequentially assigned subject number. The handling of personal data complies with the Dutch Personal Data Protection Act (De Wet Bescherming Persoonsgegevens, WBP). The SPIRIT checklist and figure for this study protocol are shown in Additional files 1 and 2.

**Table 1:** Study flow chart (except laboratory assessments)

	Screening	Treatment phase				End of Treatment	Follow-up^5^
Assessment/Evaluation	- 10 days (Day -10 to Day 0)	Week 0 Baseline/ Day 1	Completion of baseline infusion (Day 2, 3 or 4)^3^	3-6 Days after completion of baseline infusion^4^	Week 3,6,9 (± 3 days)	Week 12 (± 3 days)	Month 4, 5, 6
Informed Consent	X						
Medical History/physical examination	X						
Nerve Conduction Studies	X						
Skin biopsy and QST	X						
Laboratory Assessments (see Table 2)	X	X	X	X	X	X	
PI-NRS	X				X	X	X
PGIC					X	X	X
SFN-SIQ	X				X	X	X
NPS	X				X	X	X
SFN-RODS	X				X	X	X
Pain relief					X	X	X
Sleep quality	X				X	X	X
SF-36	X				X	X	X
Study Medication Infusion^1^		X			X		
Vital Signs^2^		X			X	X	
Concomitant Medication	X	X	X	X	X	X	X
Adverse Events	X	X	X	X	X	X	X

^1^ Medications given over 2 consecutive days at baseline and over 1 day every 3 weeks thereafter. Treatment is allowed to be prolonged up to 4 or 2 days respectively for reasons of tolerability

^2^ Vital signs (blood pressure and heart rate) to be taken right before infusion, 30 minutes after starting infusion, and immediately after infusion completed

^3^ Visit to be conducted after completion of entire baseline infusion (Day 2, 3, or 4 depending on duration of baseline infusion)

^4^ Visit to be conducted 3-6 days after completion of the baseline infusion, not 3-5 days after baseline/Day 1 infusion began

^5^ The follow-up period will be performed by standardized telephone call interviews

QST = quantitative sensory testing, PI-NRS = pain intensity numerical rating scale, PGIC = patients’ global impression of change, RT-SFN-SIQ = Rasch-transformed small fiber neuropathy symptoms inventory questionnaire, NPS = neuropathic pain scale, SFN-RODS = small fiber neuropathy Rasch-built overall disability outcome scale, SF-36 = short form 36 health survey

**Table 2:** Study flow chart – laboratory assessments

	Screening	Treatment phase				End of Treatment	Follow-up^5^
Lab assessment	- 10 days (Day -10 to Day 0)	Week 0 Baseline/ Day 1	Completion of baseline infusion (Day 2 or 3 or 4)	3-6 Days after completion of baseline infusion	Week 3,6,9 (± 3 days)	Week 12 (± 3 days)	Month 4, 5, 6
Immunofixation	X						
Pregnancy Test (Serum β HCG)	X						
TSH^1^ / regular T4	X						
Fasting blood glucose, vitamin B12^2^	X						
Serum Retain^3^	X					X	
Urinalysis	X			X			
IgG^4^		X	X^4^	X	X	X	
Hematology/CBC (hematocrit, hemoglobin, WBC, RBC, platelets)	X	X		X	X	X	
Creatinine, Blood urea nitrogen	X	X		X	X	X	
AST/ALT, LDH, potassium, bilirubin, CK		X	X^4^		X	X	
Gamma-GT		X	X^4^	X		X	

^1^ TSH to be conducted at screening if results not available since SFN diagnosis. Regular T4 automatically run by LabCorp if TSH determined to be above the upper limits of normal

^2^ To be conducted at screening if results not available since SFN diagnosis

^3^ 2 aliquots required at screening (one for viral retain and one for possible future antibody testing); 1 aliquot required at both Week 12 (for possible future antibody testing)

^4^ Samples to be obtained immediately after completion of entire baseline infusion. If the entire baseline infusion is completed in 2 days, then samples are to be collected post-infusion on Day 2. If the entire baseline infusion is completed in 3 or 4 days, then samples are to be collected post-infusion on Day 3 or 4 respectively

^5^ The follow-up period will be performed by standardized telephone call interviews

#### Adverse events

Adverse events will be recorded and monitored. The principal investigator will be informed immediately in case of any serious adverse event (SAE) occurring. Every SAE will be reported to the Ethics Committee.

### Statistical analysis

#### Sample size

A 1-point change on the PI-NRS compared to baseline is considered as the minimum clinically important difference (MCID), according to the unified rule of ½ x SD and recommendations given by the IMMPACT group [38, 43]. In the placebo group, we assume a response rate of approximately 25 % in the placebo-treated group, based on a meta-analysis of the placebo effect in pain studies in which the effect varied from 7 to 37 %, and where 16 % of the patients had a pain reduction of 50 % [32]. In the IVIg treated group, we assume a response rate of approximately 60 % based on the IMMPACT criteria [38].

Fixing a one-sided alpha at 5 %, a sample size of 24 patients per treatment group would be required to show efficacy with 80 % power and an effect size of 60 % between the two groups (chi-square test). Accounting for a dropout rate of approximately 20 % (six patients), 30 subjects per treatment group will be included in this study.

#### Type of analysis

The primary efficacy comparison is the comparison of the proportion of responders in the per-protocol population, where a responder is defined as ≥ 1-point improvement in the mean weekly peak pain measured with the PI-NRS (maximum pain) during the 12-week treatment period after first study drug infusion compared to baseline, using Kaplan-Meier curves (log rank test). The following sensitivity analyses will be performed. The primary efficacy analysis will be repeated in the per-protocol population using a more strict definition of a responder: a “responder of ≥ 2 points” is defined as ≥ 2 points improvement in the PI-NRS at the last evaluation after the first study drug infusion during the blinded 12-week treatment period compared to baseline.

In the intention-to-treat (ITT) population, the following additional sensitivity analyses will be performed: any subject who drops out with at least the week 6 PI-NRS assessment with their last mean weakly peak pain on the PI-NRS will be carried forward. A subject dropping out before the week 6 PI-NRS assessment or any subject with no baseline PI-NRS will be counted as a nonresponder independent of the last PI-NRS. Furthermore, in the ITT population, the following sensitivity analyses will be performed. The analysis will be repeated using a more strict definition of a responder: a “responder of ≥ 2 points” is defined as ≥ 2 points improvement in the PI-NRS at the last evaluation following the first study drug infusion during the blinded 12-week-treatment period compared to baseline. Subjects with no baseline or no postbaseline PI-NRS assessment will be counted as a nonresponder in this analysis.

When values are missing, we will use multiple imputation based on a regression method, using SPSS (IBM Corp. Released 2015. IBM SPSS Statistics for Macintosh, Version 22.0. Armonk, NY: IBM Corp) or Stata (StataCorp. 2013. Stata Statistical Software: Release 13. College Station, TX: StataCorp LP).

The differences between the per-protocol analyses and the ITT analyses will give a good impression of the bias that might occur in the study. Comparing these two methods, we can make a clear picture of the two populations and can investigate the true effect of IVIg in the most reliable way.

The secondary efficacy variables will be tested for treatment group differences by analyses of covariance (ANCOVAs) with the baseline measurement as the covariate and the difference of the last post-baseline measurement in the treatment period relative to baseline as an independent variable (treatment group as fixed factor). If no postbaseline measurement is documented, the baseline measurement will be used as the last postbaseline measurement. This analysis is an endpoint analysis using the “last observation carried forward” (LOCF) approach.

## Discussion

In this study, the efficacy and safety of IVIg is evaluated in patients with skin-biopsy-proven idiopathic SFN. This will be the first randomized, placebo-controlled, double-blind, clinical trial with IVIg versus placebo in patients with SFN.

In previous case studies with patients diagnosed with SFN and an underlying autoimmune disease (such as sarcoidosis, Sjögren’s syndrome, and celiac disease), IVIg has shown to be effective on chronic pain [20, 21, 24]. In addition, in chronic refractory pain in general, pain reduction after IVIg treatment has been described [23], suggesting that immunological mechanisms may play a role in the development or maintenance of pain, even if no clear immunological disorders are present.

One of the limitations of the study is that the specific mechanism of action of IVIg is not known, making it hard to predict which patients will benefit from the treatment. A second limitation might be that the treatment period of 3 months is too short to obtain effect. Third, patients will be kept stable on their current pain treatment, which could influence the results. However, stopping current pain medication would be ethically difficult.

In SFN, a better treatment is warranted because currently available (neuropathic) pain medication does not relieve pain substantially and often has side effects [44]. For IVIg, both a complementary and diminishing action on the immune system has been described [45]. In SFN, an activated immune system probably causes inflammatory responses to the small nerve fibers, which can be diminished by IVIg.

Positive findings of IVIg treatment in SFN will result in a new treatment option and may support an immunological role in this condition.

### Trial status

Participant recruitment will start at the half of 2016.

## Abbreviations

Bw, body weight; DSIS, Daily Sleep Interference Scale; eCRF, electronic case report file; GCP, good clinical practice; ICH, International Council for Harmonisation; IENFD intraepidermal nerve fiber density; IMMPACT, Initiative on Methods, Measurements and Pain Assessment in Clinical Trials; I-SFN, idiopathic small fiber neuropathy; ITT, intention-to-treat; IVIg, intravenous immunoglobulin; LOCF, last observation carried forward; MCID, minimum clinically important difference; NPS, Neuropathic Pain Scale; PGIC, Patients’ Global Impression of Change; PI-NRS, Pain Intensity Numerical Rating Scale; RT-SFN-SIQ, Rasch-Transformed Small Fiber Neuropathy Symptoms Inventory Questionnaire; SAE, serious adverse event; SF, 36 Short Form 36 Health Survey; SFN, small fiber neuropathy; SFN-RODS, small fiber neuropathy Rasch-built Overall Disability Outcome Scale; WBP, Wet Bescherming Persoonsgegevens

## Additional files

Additional file 1: SPIRIT 2013 Checklist: recommended items to address in a clinical trial protocol and related documents*. (DOC 121 kb) Additional file 2: Example template of recommended content for the schedule of enrolment, interventions, and assessments*. (DOC 68 kb)
