# Supplementary material for: Intravenous immunoglobulin therapy for small fiber neuropathy: study protocol for a randomized controlled trial
Source: Trials. 2016 Jul 20;17:330. doi: 10.1186/s13063-016-1450-x (PMC4955261; doi:10.1186/s13063-016-1450-x)
Supplement: Additional file 2: — Example template of recommended content for the schedule of enrolment, interventions, and assessments*. (DOC 68 kb) [file 13063_2016_1450_MOESM2_ESM.doc]

Additional file 2. Example template of recommended content for the schedule of enrolment, interventions, and assessments.*

|  | **STUDY PERIOD** | | | | | | | |
| --- | --- | --- | --- | --- | --- | --- | --- | --- |
|  | **Enrolment** | **Allocation** | **Post-allocation** | | | | **End of treatment** | **Follow-up** |
| **TIMEPOINT**** | ***-t_1_*** | **0** | ***v_1_*** | ***v_2_*** | ***v_3_*** | ***v_4-6_*** | ***v_7_*** | ***v_8_-_10_*** |
| **ENROLMENT:** |  |  |  |  |  |  |  |  |
| **Eligibility screen** | X |  |  |  |  |  |  |  |
| **Informed consent** | X |  |  |  |  |  |  |  |
| **Medical history** | X |  |  |  |  |  |  |  |
| **Physical examination** | X |  |  |  |  |  |  |  |
| **Nerve conduction studies** | X |  |  |  |  |  |  |  |
| **Skin biopsy and QST** | X |  |  |  |  |  |  |  |
| **Allocation** |  | X |  |  |  |  |  |  |
| **INTERVENTIONS:** |  |  |  |  |  |  |  |  |
| ***Intravenous Immunoglobulin*** |  |  |  |  |  |  |  |  |
| ***Placebo*** |  |  |  |  |  |  |  |  |
| **ASSESSMENTS:** |  |  |  |  |  |  |  |  |
| **Laboratory assessments** | X |  | X | X | X | X | X |  |
| ***PI-NRS*** | X |  |  |  |  | X | X | X |
| ***PGIC*** |  |  |  |  |  | X | X | X |
| ***SFN-SIQ*** | X |  |  |  |  | X | X | X |
| ***NPS*** | X |  |  |  |  | X | X | X |
| ***SFN-RODS*** | X |  |  |  |  | X | X | X |
| ***Pain relief*** |  |  |  |  |  | X | X | X |
| ***Sleep quality*** | X |  |  |  |  | X | X | X |
| ***SF-36*** | X |  |  |  |  | X | X | X |
| ***Study medication infusion*** |  |  | X |  |  | X |  |  |
| ***Vital signs*** |  |  | X |  |  | X | X |  |
| ***Concomitant medication*** | X | X | X | X | X | X | X | X |
| ***Adverse events*** | X | X | X | X | X | X | X | X |

*Recommended content can be displayed using various schematic formats. See SPIRIT 2013 Explanation and Elaboration for examples from protocols.

**List specific timepoints in this row.

v1 = Week 0 baseline / day 1

v2 = Completion of baseline infusion

v3 = 3-6 days after completion of baseline infusion

v4 = week 3 (second treatment)

v5 = week 6 (third treatment)

v6 = week 9 (fourth treatment)

v7 = week 12 (end of treatment)

v8 = month 4 (follow-up)

v9 = month 5 (follow-up)

v10 = month 6 (follow-up)
